# Measurement of Saccade Parameters in Relation to Adaptation to Homonymous Hemianopia

**DOI:** 10.22599/bioj.272

**Published:** 2022-09-28

**Authors:** Claire Howard, Paul Knox, Helen Griffiths, Fiona Rowe

**Affiliations:** 1 Salford Care Organisation, Northern Care Alliance NHS Foundation Trust, GB; 2 University of Liverpool, GB; 3 Sheffield Children’s NHS Foundation Trust, GB

**Keywords:** saccades, hemianopia, saccadometer, stroke, adaptation, calibration

## Abstract

**Purpose:**

To report saccade parameters in participants during adaptation to post-stroke homonymous hemianopia.

**Methods:**

In a prospective observational case cohort study, adult stroke survivors with new onset homonymous hemianopia were recruited. Using quantitative measurement, saccade parameters were measured and compared between the hemianopic and non-hemianopic sides. Two participants with longitudinal measurements were compared with age-matched controls.

**Results:**

Of 144 clinical study participants, quantitative saccade measurements were only possible in 14 due to an inability to visualise targets on the hemianopic side in the majority. In 9 of the 14 participants, at four weeks post-stroke, mean (±SD) saccade latency was significantly longer to the hemianopic (328.4 ± 105.9 ms) compared to the non-hemianopic side (234.7 ± SD53.6 ms; *t* = 4.2, *df* = 8, *p* = 0.003). The number of correct saccadic responses out of 50 was significantly lower to the hemianopic side (36.6 ± SD14.1) in comparison to the non-hemianopic side (44.4 ± SD7.5; *t* = –3.1, *df* = 8, *p* = 0.014). In two participants studied over an eight-week time period, saccadic differences to the hemianopic side persisted despite apparent recovery of visual field.

**Conclusion:**

As participants with residual visual field loss were unable to perform quantitative assessments, the widespread use of this approach in this setting is limited. However, in those whom measurements were possible, there were statistically significant differences in saccade parameters between hemianopic and non-hemianopic sides that persisted post-visual recovery. Exploration of saccades in relation to adaptation to hemianopia and response to saccadic scanning/search training requires further examination.

## Introduction

One visual consequence of stroke is homonymous hemianopia with a reported point prevalence of 28% (Rowe et al. 2019). People with hemianopic field defects cannot process the visual world in the same way as those with a full visual field. They have difficulty in detecting and locating objects in the visual space to the affected side. A hemianopic visual field defect often leads to difficulty with viewing a scene with enough speed to make sense of the scene as a whole (Pambakian & Kennard 1997).

The primary aim of the overall research study was to investigate the factors important for adaptation to post-stroke homonymous hemianopia (Howard et al. 2021). As a secondary objective, we investigated saccade parameters of participants recruited to this UK-based clinical study. Hemianopia is reported to alter saccades, and a change in saccadic behaviour could be an important factor for the adaptation process.

Saccades are fast eye movements that move both eyes in the same direction quickly, with the aim of placing the image of a fixated object of interest onto the fovea (Leigh & Zee 2015). There are several subtypes of saccades that involve different brain areas and cognitive functions. Saccades can be reflexive or voluntary and are closely related to cognitive processes such as inhibition, memory and attention. Their assessment can help provide information regarding the dysfunction of affected areas in the brain. These saccadic subtypes can be described within a hierarchy of eye movements (Wong 2008). At the lowest end of this hierarchy are the automatic eye movements that reset the eyes following spontaneous drifting, such as in the quick phase of optokinetic nystagmus. At the next level are saccades guided by vision, known as reflexive saccades. These are generated in response to visual or auditory onsets and often constitute an orientation reflex. Higher-level saccades are known as volitional or voluntary saccades (Wong 2008). This research focused on the measurement of visually guided reflexive saccades which can be described rigorously using the main sequence parameters of latency, duration, peak velocity and amplitude (Leigh & Kennard 2004). These saccadic parameters can be measured in a quantitative manner using a variety of methods.

Measuring saccades in hemianopia brings its own limitations and considerations. It is often unclear whether an individual has impaired saccades due to the lack of visual information related to the target within the hemianopic field, or an underlying neurological saccadic deficit, or a combination of these factors.

Previous studies have been able to measure saccades during visual search in homonymous hemianopia. Zihl examined scanning behaviour in 60 hemianopic patients using infraed video recordings (Zihl 1995). During this study, a special calibration procedure was utilised whereby subjects’ position of gaze was continuously calculated by tracking corneal reflections with respect to the centre of the pupil. During this calibration, the subject’s head was fixed into position using head restraints. This study concluded that some patients show successful oculomotor adaptation to their hemianopia, whereas others remain impaired. Those with impaired scanning may have damage to the brain areas that also contribute to the control of saccades. Furthermore, the author argues that there is potential to improve quality of life with specific training (Zihl 1995).

Pambakian et al. also used infrared video recordings to explore the way in which eight patients with homonymous hemianopia scanned the world. They reported that patients made significantly more saccades towards their hemianopic side, with these saccades being smaller in amplitude and shorter in duration to those of the seeing side (Pambakian et al. 2000). This saccadic compensation is thought to limit an individual’s ability to effectively search their environment, contributing to obstacle avoidance problems and disorientation (Kerkhoff 1999). Kerkoff concluded that the eye movement changes observed could be due to the development of compensatory eye movement strategies employed by those with hemianopia. In other words, individuals with hemianopia tended to undershoot the target to a greater extent than those with a full visual field and hence required more saccades to reach their intended target.

Both studies involved the use of chin rests and head restraints during the measurement of saccades. This limits the feasibility of replicating such measurements in an acute stroke setting on acutely unwell individuals. Although many of the participants in these two research studies had a homonymous hemianopia caused by stroke, they were not assessed at an acute stage but seen at a time period of at least three months following onset.

A further study sought to enhance understanding of target detection and saccadic responses in hemianopia (Fayel et al. 2014). This study reports that hemianopic patients retain the ability to direct a saccade to the affected side and that saccadic parameters are altered by the defect. Fayel et al. used an EyeLink video-based tracker to measure saccades. They overcame calibration issues by allowing patients to scan to fixation targets during the calibration process, meaning they were measuring voluntary saccades and not a visually directed response.

Saccadic eye movement training has been reported as a compensatory intervention method for hemianopia (Pambakian et al. 2004, Zihl 1995). The training aims to improve saccadic eye movement skills and hence reduce the demands of everyday visual tasks. Specifically, scanning training aims to improve the speed and accuracy of the detection of targets/objects to the affected hemianopic side. This improvement in speed and accuracy of object detection has the potential to improve adaptation.

A Cochrane systematic review reported that there was some evidence that this type of compensatory eye movement training for hemianopia could be beneficial in improving quality of life. However, there was some evidence that scanning training has no effect on other outcomes, including extended activities of daily living, reading, visual field measurements and scanning ability (Pollock et al. 2019).

Therefore, in this study we explored the relationship between saccades to the hemianopic and non-hemianopic sides and with age-matched controls. More research is needed in this area if the association between saccadic eye movement training and adaptation is to be fully understood.

## Materials and Methods

Ethical approval was obtained from the UK Health Research Authority and North West (Preston) Research Ethics Committee (16/NW/0542). Written informed consent was obtained for the assessments that were additional to a standard visual orthoptic assessment; this included the formal saccadic measurements using a saccadometer. If a participant was unable to sign the consent form due to functional disability, witnessed consent was sought.

In a prospective observational case cohort study, adult stroke survivors with new onset homonymous hemianopia were recruited. The measurement of saccade parameters using a head-mounted infrared eye tracker (Saccadometer Advanced LatencyMeter 6.5, Ober Consulting SN 1152) was attempted for all study participants as part of a comprehensive assessment of visual and navigational functions (Howard et al. 2021). Participant demographics that included age, gender, ethnicity and postcode were collected. Using the participants’ postcode, an income deprivation decile score was calculated using the Ministry of Housing, Communities and Local Government calculator (Ministry of Housing 2015). A formal quantitative measure of visual field was undertaken where possible with an automated perimeter using a binocular Esterman programme. Where formal perimetry was not possible, a standardised confrontation method was employed using both static and kinetic target presentation using a 1 cm diameter red target. Grading of visual fields was undertaken by means of calculating a percentage of visual field loss to the hemianopic and unaffected sides.

Visual attention was assessed using a combination of three paper-based tests: line bisection, clock drawing and cancellation tests. The combined results were used to make a clinical decision on the presence and extent of any visual inattention, coupled with clinical observations by the multidisciplinary team.

Paper-based scanning exercises were offered to all participants as standard. These exercises were aimed at training a participant to adapt to their vision loss and were piloted in the VISION trial (Rowe et al. 2016).

The saccadometer was selected as the measurement device of choice. The saccadometer uses infrared oculography to measure binocular eye movements in the horizontal plane and has been reported as an accurate and non-invasive method for saccade measurement (Bargary et al. 2017). It is fully portable and has the advantage of having no requirement to stabilise the head during measurements. As the infrared stimuli are head mounted and therefore move with the participant’s head, it was not necessary to fix the head position during testing. However, participants were asked and encouraged to keep their heads as still as possible when the measurements were taking place.

Participants were exposed to 100 horizontal step trials in which targets were presented 10° from fixation at a distance of 1 m. Step trials consist of a simple 10° step task, with central fixation at the start of each trial. The duration of the central fixation target (range 1–2 seconds) and direction were randomised, as described in Table 1 and taken from the Ober Consulting User Guide (Ober Consulting 2014). The horizontal target was extinguished when the eye was detected as having moved towards it.

**Table 1:** Step trial sequence.


PHASE DESCRIPTION	STIMULI	PHASE NAME

1.Central fixation	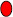	Randomised foreperiod*

2.Left or right, randomised direction	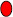	Left target

	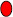	Right target

Repeat steps 1–2 until pre-set number of trials is recorded		


* *Foreperiod* refers to the time delay between the central target display and the left/rightward target display.

Eye position was sampled at a speed of one kilohertz (kHz), which provided a temporal resolution of 1 ms (Ober Consulting 2014). The participant was positioned in a fixed chair at a distance of 1 m in front of a matt white wall.

Following saccadic eye movement recordings, data were downloaded from the saccadometer to a computer using the LatencyMeter application (LatencyMeter 2016). This application software supports automatic measurements of saccade trials and statistical measures. For each valid trial, saccade amplitude, peak velocity, duration and latency were available. Measurements were attempted at baseline (within four weeks of stroke onset), and at 4, 12 and 26 weeks post-stroke.

## Control saccade measurements

Age, gender and general demographic matched controls were used to collect data under the same conditions, following the same procedures as those used for stroke patients. Control data was collected following written consent and at one time point.

The matching was performed against the five stroke patients who had measurable saccadic eye movement data at repeated time points. Control participants were screened for ocular and general history to ensure there was no significant history of eye conditions and/or stroke.

## Limitations of saccadometer

The study plan was to measure eye movements using a saccadometer in all participants to assess saccadic parameters to each side and to monitor any changes in these measurements throughout the adaptation journey. During the early stages of study development, it became apparent that saccades cannot be measured in this way for the majority of participants due to a complete homonymous hemianopia and their inability to see the visual targets on the hemianopic side. The saccadometer requires an individual to look towards the visual targets on the right and left sides to allow calibration of the instrument. *Calibration* is defined as the process of mapping eye tracker measurements to physical gaze direction or gaze position (Holmqvist et al. 2022). To extract saccade parameters, all eye tracker systems require calibration that entails having the participant accurately direct their eyes at an external target whose position is known. Due to natural variability in saccade endpoints, this has to be repeated for individual target positions and the results averaged. When working with clinical participants, with either visual impairments or peripheral/central pathology of the oculomotor system, this calibration becomes highly problematic. For example, inaccuracies would be created if calibrations were to be derived from the non-hemianopic side to the hemianopic side, particularly where the deficits in question are neurological rather than, for example, retinal.

In summary, calibration of the instrument was not possible for those recruits with a complete macular splitting homonymous hemianopia. The study’s methodology was adapted to only include saccadic measurements with those participants with a partial hemianopia: those who were able to visualise the target on both sides and hence move their eyes to fixate the targets. Calibration of the saccadometer was attempted for all participants who demonstrated at least 10° of central visual field on their hemianopic side (a partial hemianopia). If they were unable to visually locate the calibration target and calibration was not possible, the measurement was abandoned on the day and attempted at the next routine assessment. The success of calibration was not dependent on the total percentage of visual field loss to the hemianopic side; rather, the participants’ ability to locate the target in the precise location (10° right and left of central fixation) was required for calibration to occur.

## Data Analysis

Saccade parameter data were summarised using means and standard deviations. Prior to any data analysis, incorrect trials were filtered out and removed. Saccades with a latency less than 50 ms were excluded, in line with other saccade research (Krismer et al. 2010; Knox, Wolohan & Helmy 2017; Amatya, Gong & Knox 2011). Saccades with latencies this low are generated too fast to be a response to the visual stimulus and are classed as anticipatory.

Saccade data for all collected measurements was divided into hemianopic and non-hemianopic sides. For each participant, mean saccade latency, peak velocity and amplitude were calculated for targets directed towards the hemianopic side and the non-hemianopic side. The same analysis was applied to age-matched control data, with comparisons made between rightward and leftward saccades. A mean calculation of the proportion of correct responses to each side (out of a possible 50) was also made. A correct response was a response where a saccadic latency of ≥50 milliseconds was recorded. Paired sample t-tests were used to compare mean scores between the two sides, using the four-week measurement if available.

In addition, the mean and standard deviation results were used to calculate a coefficient of variation (CoV) for the saccadic parameters, comparing participants with controls for the hemianopic and non-hemianopic/right and left sides. The CoV is a statistical measure of the dispersion of data points around the mean while correcting for the size of the mean. It was calculated to show the extent of variability in the samples. For the purpose of describing the data, leftward saccades are assigned negative amplitude and rightward saccades positive amplitudes.

Two case studies with repeated measurements were explored in more detail with age-matched control data to calculate a CoV, comparing the sides and with controls.

## Results

Of 144 participants recruited to the full clinical study, quantitative visually directed reflexive saccade measurements were only possible in 14 due to an inability to visualise targets on the hemianopic side in the majority of participants. Of these 14 participants, 1 was measured at four time points, 4 at two time points and the remaining 9 at a single time point, giving a total of 21 measurements overall. Table 2 provides an overview of the time points at which measurements were possible.

**Table 2:** Measurement overview for participants (n = 14).


PARTICIPANT ID	BASELINE	4 WEEKS	8 WEEKS	12 WEEKS	26 WEEKS	TOTAL

1	X	✔	X	X	X	1

2	X	✔	X	X	✔	2

3	X	✔	X	X	X	1

4	X	X	X	✔	X	1

5	X	✔	✔	X	X	2

6	X	✔	X	X	X	1

7	X	✔	✔	X	X	2

8	X	✔	X	X	X	1

9	X	X	X	✔	✔	2

10	X	✔	X	X	X	1

11	X	X	X	✔	X	1

12	X	X	X	✔	X	1

13	X	X	X	✔	X	1

14	X	✔	✔	✔	✔	4


Of the assessed 14 participants, there were 11 males (78.6%) and 3 females (21.4%), with a mean age of 61.2 (SD 13.3) years. All 14 were of white British ethnicity, and all had suffered an ischaemic stroke. Table 3 outlines demographic and characteristic data for assessed participants in comparison to the 130 who were not assessed. There were no statistically significant differences in characteristics found between the two groups. The group of participants assessed showed a lower total percentage loss of visual field on the hemianopic side, which was expected as only partial hemianopia could be assessed. However, this difference was not statistically significant. None of the participants undergoing saccadic measurements had any evidence of visual inattention.

**Table 3:** Demographics and characteristics: saccades tested or not tested.


		TESTED n = 14	NON-TESTED n = 130	*p* value

Age (years)	Mean (SD)	61.2 (13.3)	67.8 (12.3)	0.930

Gender	Male (%)	11 (78.6)	88 (67.7)	0.253

	Female (%)	3 (21.4)	42 (32.3)	

Deprivation score	Mean (SD)	5.8 (3.1)	4.7 (3.0)	0.953

Side of hemianopia	Right (%)	6 (42.9)	63 (48.5)	0.860

	Left (%)	8 (57.1)	67 (51.5)	

Total % visual field loss at baseline	Mean (SD)	48.2 (19.6)	75.8 (23.4)	0.056


To compare saccadic parameters, Table 4 displays data for all recorded trials recorded at the four-week review for saccades to the hemianopic side and to the non-hemianopic side for comparison. All measurements were recorded with both eyes open (binocularly). There were nine participants who provided data at the four-week time point (Table 2). This time point was selected as there is a measurement for the majority of participants and recovery of visual field is minimal at this time point.

**Table 4:** Step saccade data comparison: hemianopic side against non-hemianopic side. * Significant result.


		TOWARDS HEMIANOPIC SIDE n = 9	TOWARDS NON-HEMIANOPIC SIDE n = 9	*p* VALUE	*t* VALUE	*df* VALUE

Step saccadic latency (ms)	Mean (SD)	328.4 (105.9)	234.7 (53.6)	0.003*	4.2	8.0

Step saccadic peak velocity (°/s)	Mean (SD)	422.5 (98.0)	461.8 (126.4)	0.468	–0.8	8.0

Step saccadic amplitude (°)	Mean (SD)	10.4 (2.6)	14.5 (8.3)	0.165	–1.5	8.0

Number of correct responses (/50)	Mean (SD)	36.6 (14.1)	44.4 (7.5)	0.014*	–3.1	8.0


Statistically significant differences were evident between the two sides. Saccadic mean latencies were longer to the hemianopic side (328.4 ± SD105.9 ms) compared to the non-hemianopic side (234.7 ± SD53.6 ms), with this difference being statistically significant (*t* = 4.2, *df* = 8, *p* = 0.003). There was a difference in mean peak velocity of saccades between the hemianopic (422.5 ± SD98.0 °/s) and non-hemianopic sides (461.8 ± SD126.4 °/s). However, this difference was not statistically significant (*t* = –0.8, *df* = 8, *p* = 0.468). In addition, for saccades to the hemianopic side, saccadic amplitude was lower than to the non-hemianopic side 10.4 ± SD2.6° vs 14.5 ± 8.3°, but the difference in amplitude was not significant (*t* = –1.5, *df* = 8, *p* = 0.165). The difference seen in peak velocities can be explained by the difference in saccadic amplitudes, as these measurements are directly related. The mean number of correct saccadic responses out of a possible 50 was significantly lower towards the hemianopic side (36.6 ± SD14.1) in comparison to the non-hemianopic side (44.4 ± SD7.5; *t* = –3.1, *df* = 8, *p* = 0.014).

These differences in saccadic parameters were evident between the hemianopic and non-hemianopic sides despite the participants being able to view the target based on the partial nature of their visual field loss. Calibration of the saccadometer was only possible if the participant was successfully able to visually locate the horizontal target positioned at 10° to the right or left of central fixation and generate an eye movement to it.

## Case Study Presentation

To explore potential changes in saccades over time, the data from two participants were analysed in more detail (participants 5 and 14 from Table 2). Longitudinal data were available from them, and both exhibited a complete recovery of visual field loss as determined by binocular Esterman visual field testing. These cases demonstrated that, in general, performance to the hemianopic side was more variable in patients than controls and that saccadic differences to the hemianopia side remained despite recovery of visual field.

## Case study one (CS1)

The first participant analysed (participant 5 in Table 2) was a white British male aged 67 years at the time of his stroke. The diagnosis of occipital ischaemic stroke was made from clinical presentation, as his acute presentation CT brain scan was reported as normal and no further brain scans were considered clinically necessary. Two saccadic measurements were possible for this participant at four weeks and eight weeks post-stroke. He had a full recovery of Esterman visual field (eight-week visual field loss 0.0%), meaning he did not wish to attend clinic for further follow-up assessments after his eight-week visit.

### CS1 baseline results

**Figure 1:**
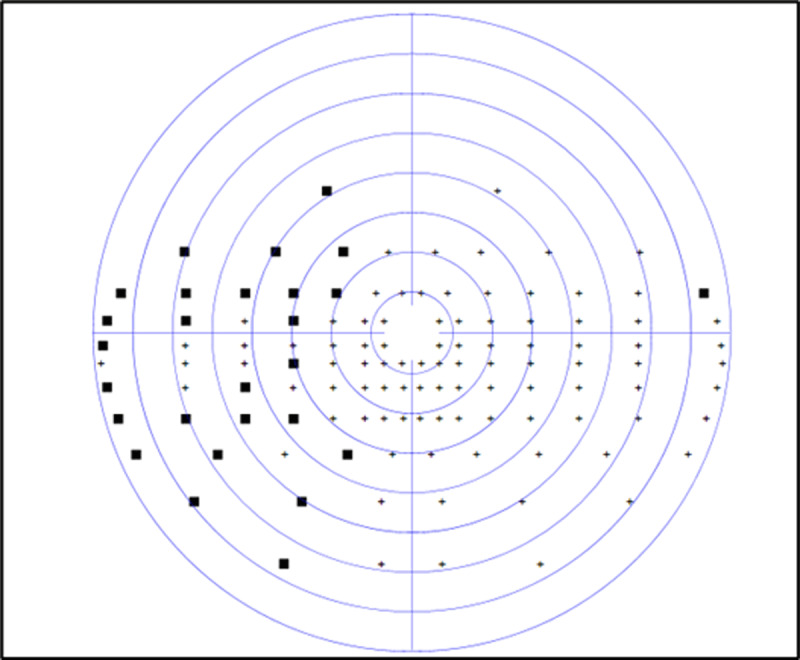
CS1 baseline binocular Esterman visual field result. missed target ■.

At baseline assessment, the participant was graded as having a partial left-sided hemianopic visual field loss of 43.3%. Figure 1 displays his binocular Esterman visual field results (Octopus perimeter) at baseline (five days post-stroke). At the time of baseline assessment, saccadic measurements were not possible, as he was unable to visualise the calibration target on the affected left side.

### CS1 four-week results

**Figure 2:**
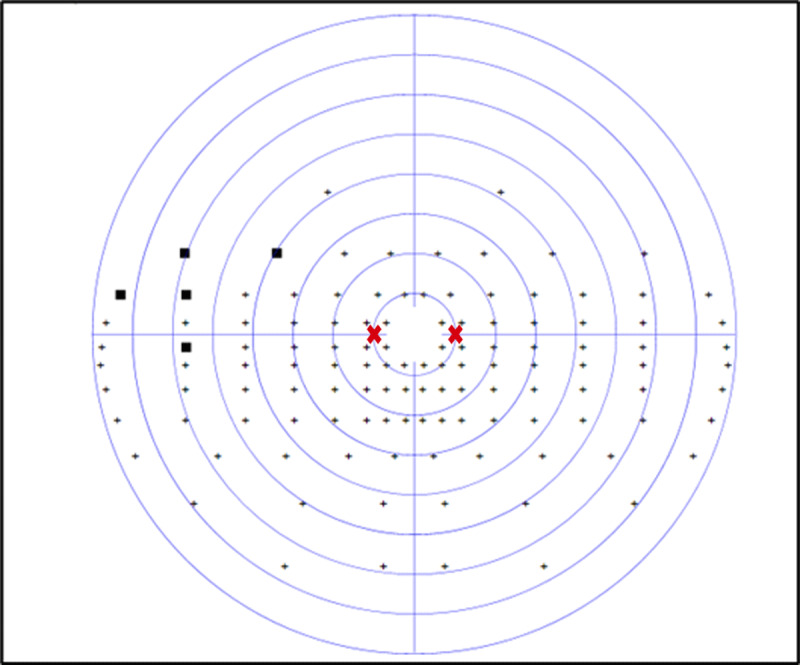
CS1 four-week binocular Esterman visual field result. Missed target ■ Saccade target position 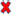.

At the four-week assessment, he displayed significant improvements in his initial partial left hemianopic visual field loss, with only 5 missed targets out of 60 on the previously hemianopic side. The hemianopia had improved to a mainly superior quadrantanopic defect, graded at 8.3%. Figure 2 displays his visual field assessment, which shows the area of visual field recovery to include 10° to the left of central fixation, resulting in the participant being able to visualise the calibration and test targets. Since his baseline assessment, he had undertaken visual scanning exercises daily for at least 30 minutes, which involved practising saccadic eye movements.

Although his visual field loss was only minimal at this stage, saccadic parameters to the left hemianopic side showed differences to the right side. Mean amplitude of leftward saccades was 7.6° (SD 1.1°) compared to 10.5 (SD 1.2°) towards the right (*p* < 0.001). This difference is shown in Figure 6, which displays the amplitude/peak velocity plot at four weeks in comparison to a normally sighted age-matched control participant.

A coefficient of variation (CoV) calculation displays this variance in dispersion (Table 5). Participant CS1 showed slightly more variation that the control subject for saccades to both sides.

**Table 5:** Coefficient of variation (CoV) calculations for CS1 and age-matched control at four weeks and eight weeks.


		CS1 HEMIANOPIC SIDE	CS1 NON-HEMIANOPIC SIDE	CONTROL RIGHT SIDE	CONTROL LEFT SIDE

CoV % saccadic amplitude	Four weeks	14.5	11.4	7.8	9.2

	Eight weeks	15.9	10.4		

CoV % saccadic velocity	Four weeks	7.9	9.2	6.0	7.9

	Eight weeks	11.6	9.0		


### CS1 eight-week results

**Figure 3:**
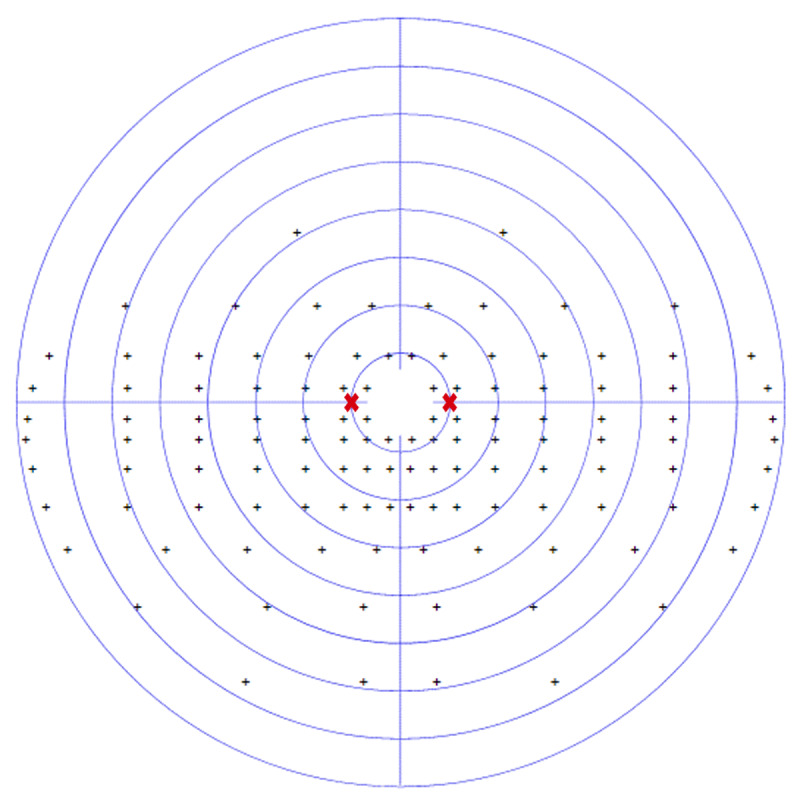
CS1 eight-week binocular Esterman visual field result. Saccade target position 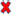.

**Figure 4:**
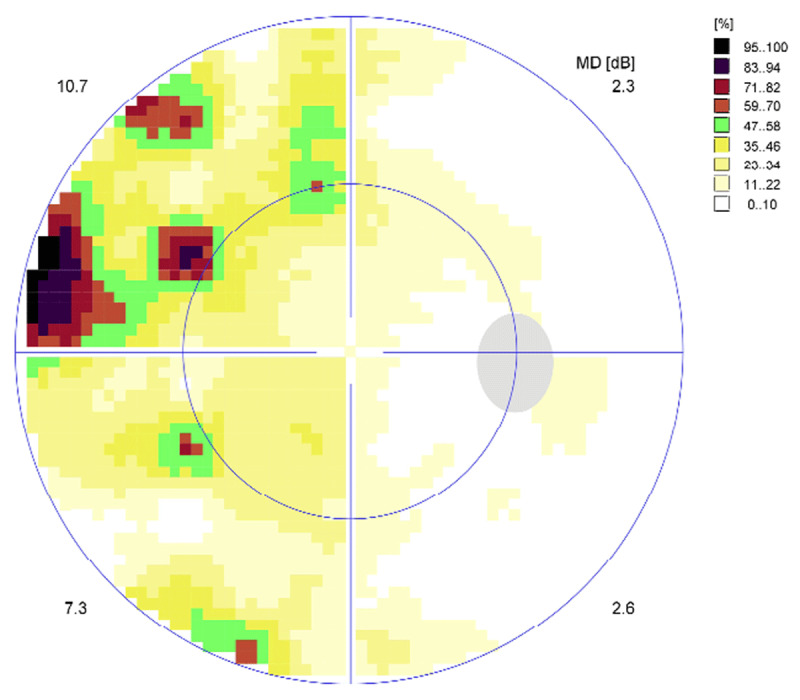
CS1 eight-week right eye threshold visual field test result. Representation of the central 30° of visual field area at eight weeks post-stroke.

**Figure 5:**
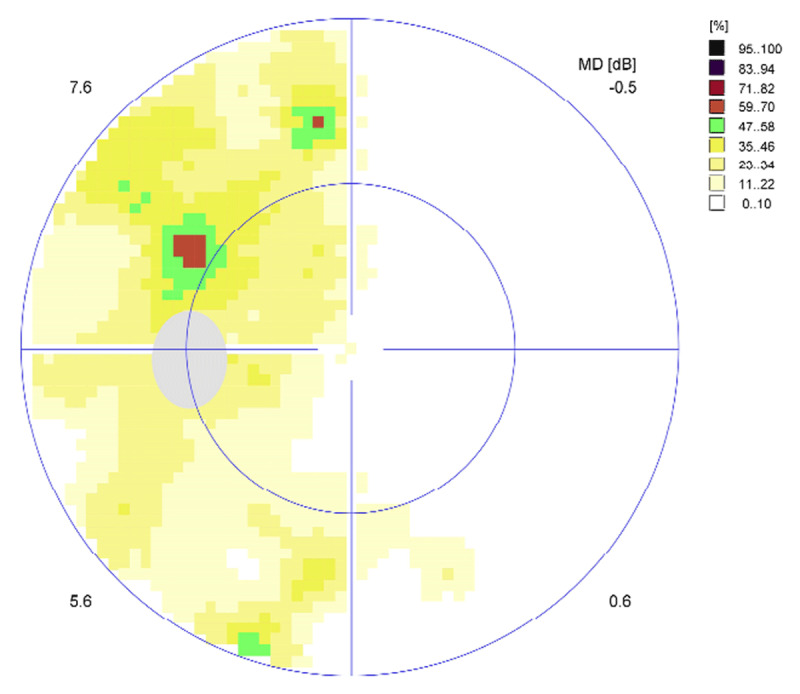
CS1 eight-week left eye threshold visual field test result.

At eight weeks CS1 demonstrated further improvement in his visual field with a completely normal visual field result on Esterman perimetry testing (0.0% loss) (Figure 3). On the same day, this participant underwent threshold perimetry testing to ascertain any relative visual field defects that are not always detected by Esterman testing alone (Figures 4 and 5). Threshold testing demonstrated a residual visual field defect to the left side, which was particularly evident in the superior quadrant of both eyes.

Since his four-week assessment, he had continued to undertake visual scanning exercises daily for at least 30 minutes. At the eight-week assessment, saccadic parameters (Figure 7) appeared to be relatively unchanged, despite the apparent improvement in visual field loss.

**Figure 6:**
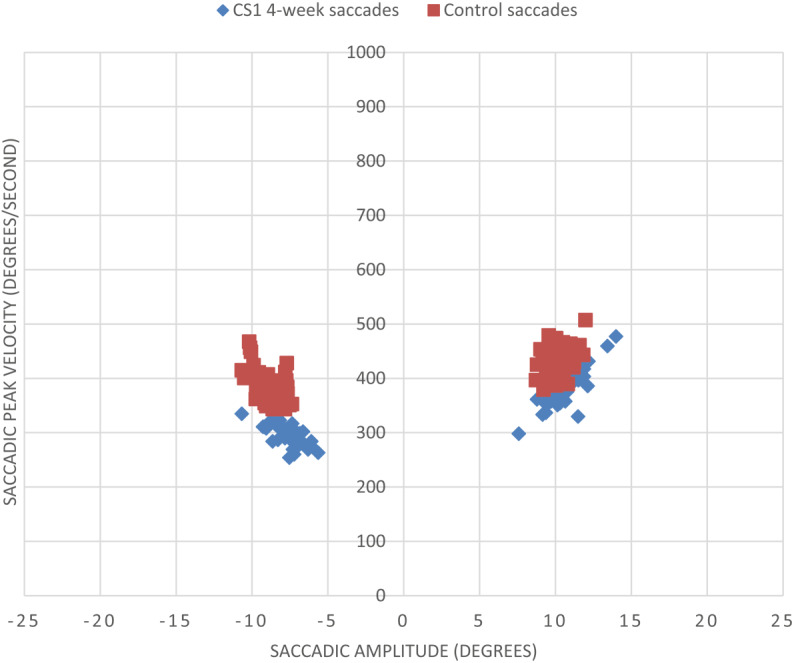
Saccadic peak velocity v saccadic amplitude for CS1 at four weeks and a control subject (minus values represent leftward saccades into hemianopic side). The control subject displayed in Figure 6 makes accurate saccades to the target, which was presented at 10° to the right and left. The peak velocity shows a small variation with clustering of measurements around the same region. In contrast, CS1 displays more variation in the peak velocity measurements of his saccades and less accuracy to locate the target at its 10° location.

**Figure 7:**
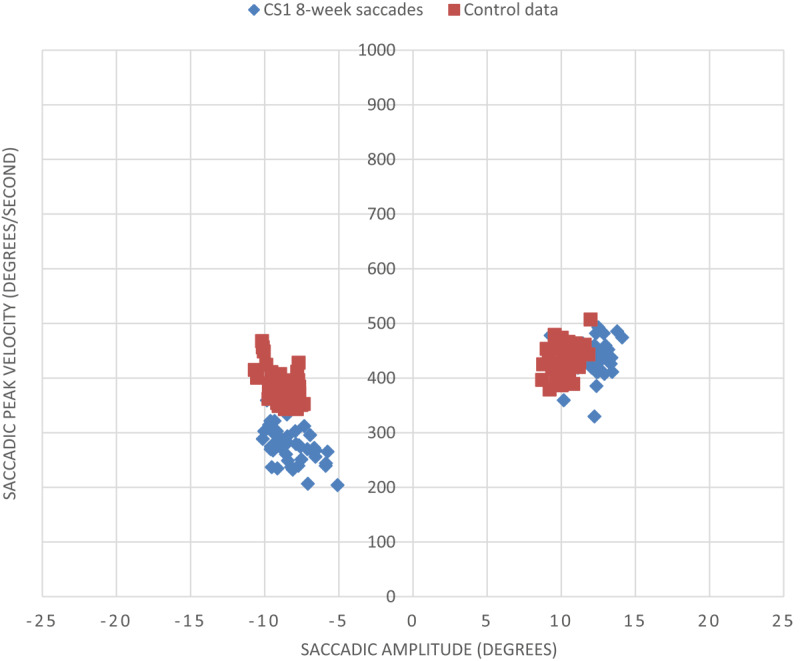
Saccadic peak velocity v saccadic amplitude for CS1 at eight weeks and a control subject (minus values represent leftward saccades into hemianopic side). There is minimal change in saccadic parameters for CS1 at eight weeks post-stroke from the four-week measurements (as displayed in Figure 6). A slight reduction in peak velocity to the hemianopic side at eight weeks post-stroke is evident, and considerable variation between the right and left sides remains.

When comparing the CoV scores from this eight-week assessment to the previous four-week assessment, there was slightly more variation on what was previously the hemianopic side (Table 5). Overall, there was more variation in scores between the right and left sides for the participant than for age-matched control, suggesting an underlying saccadic deficit remained to some extent.

## Case study two (CS2)

To explore the relationship in saccadic parameters over time, a second case was also explored (CS2, participant 14 in Table 2). The participant was a white British male aged 37 years at the time of his stroke. The diagnosis of occipital ischaemic stroke was made from clinical presentation, as his acute presentation CT brain scan was reported as normal and no further brain scans were considered clinically necessary. This participant was selected for in-depth analysis due to the multiple time points at which assessments were available. In addition, he had a full recovery of Esterman visual field (eight-week visual field loss 0.0%).

### CS2 baseline results

**Figure 8:**
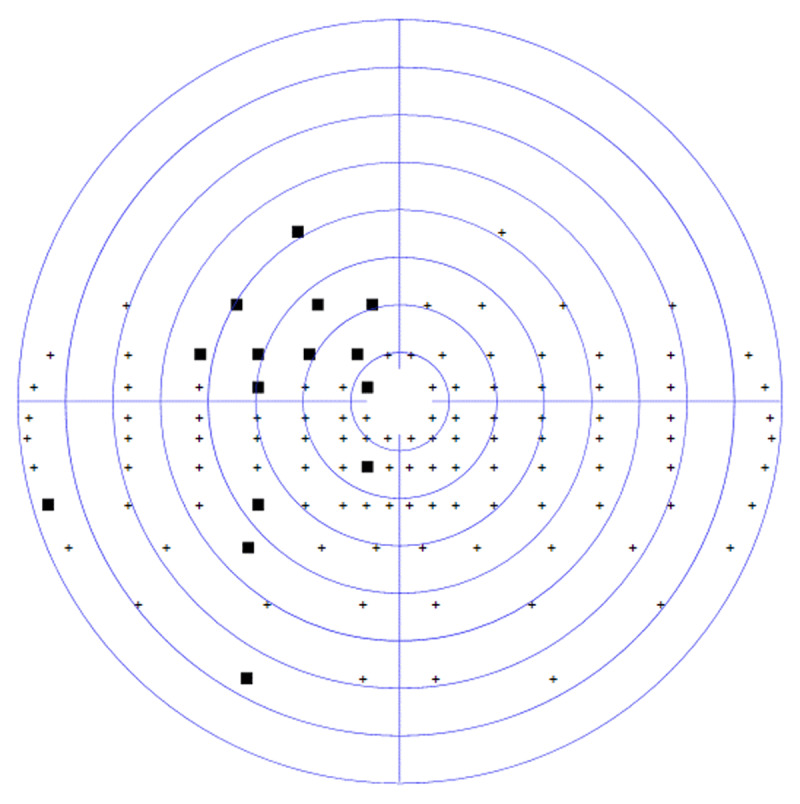
CS2 baseline binocular Esterman visual field result. Missed target ■.

At baseline assessment, CS2 was graded as having a partial left-sided hemianopic visual field loss of 25.0%. Figure 8 displays the binocular Esterman visual field results for this participant at baseline (two days post-stroke). At the time of baseline assessment, saccadic measurements were not possible, as he was unable to visualise the calibration target on the affected left side.

### CS2 four-week results

**Figure 9:**
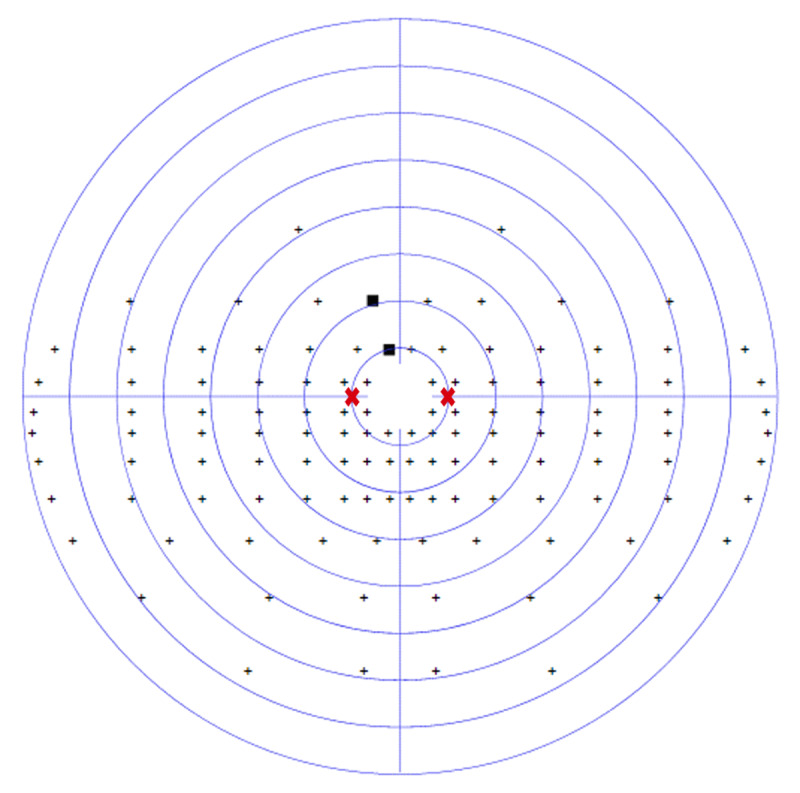
CS2 four-week binocular Esterman visual field result. Saccade target position 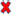.

At the four-week assessment, he displayed significant improvements in his initial partial hemianopic visual field loss, with only 2 missed targets out of 60 on the previously hemianopic side. The hemianopia had improved to a partial superior quadrantanopia, graded at 3.3%. Figure 9 displays his visual field assessment results. Since his baseline assessment, he had been undertaking visual scanning exercises daily for at least 30 minutes, which involved practising saccadic eye movements.

Despite his minimal visual field loss, saccadic parameters to the left side were affected when compared to the right side. Mean amplitude of saccades to the left side was 10.5° (SD 1.5°) compared to 8.3 (SD 2.9) on the right (*p* < 0.001).

A difference between peak velocity and amplitude for participant CS2 at four weeks in comparison to a normally sighted age-matched control subject is displayed in Figure 13. The leftward saccades of CS2 were notably more variable in both amplitude and peak velocity. A CoV calculation displays this variance in dispersion (Table 6). Participant CS2 has a CoV score of 35.6% to the previously hemianopic side, showing considerably more variation than the control subject for leftward saccades (10.5%). The control subject had a more symmetrical saccadic response to the right and left sides as displayed in Figure 13, with considerably less variation (Table 6).

**Table 6:** Coefficient of variation (CoV) calculations for CS2 and age-matched control at four weeks and eight weeks.


		CS2 HEMIANOPIC SIDE	CS2 NON-HEMIANOPIC SIDE	CONTROL RIGHT SIDE	CONTROL LEFT SIDE

CoV % saccadic amplitude	Four weeks	35.6	14.6	7.7	10.5

	Eight weeks	22.2	21.8		

CoV % saccadic velocity	Four weeks	26.5	16.0	6.3	9.0

	Eight weeks	14.0	15.1		


Participant CS2 shows a much higher level of variation for saccades towards the hemianopic than towards the non-hemianopic side, especially at four weeks post-stroke. Saccades are higher for both sides than for the age-matched control subject.

### CS2 eight-week results

**Figure 10:**
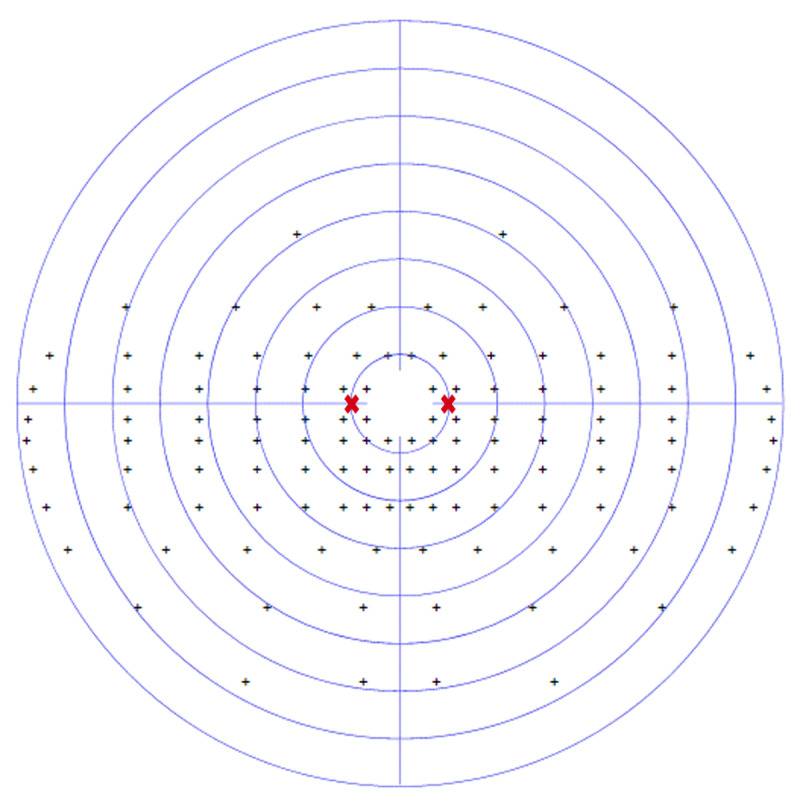
CS2 eight-week binocular Esterman visual field result. Saccade target position 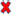.

**Figure 11:**
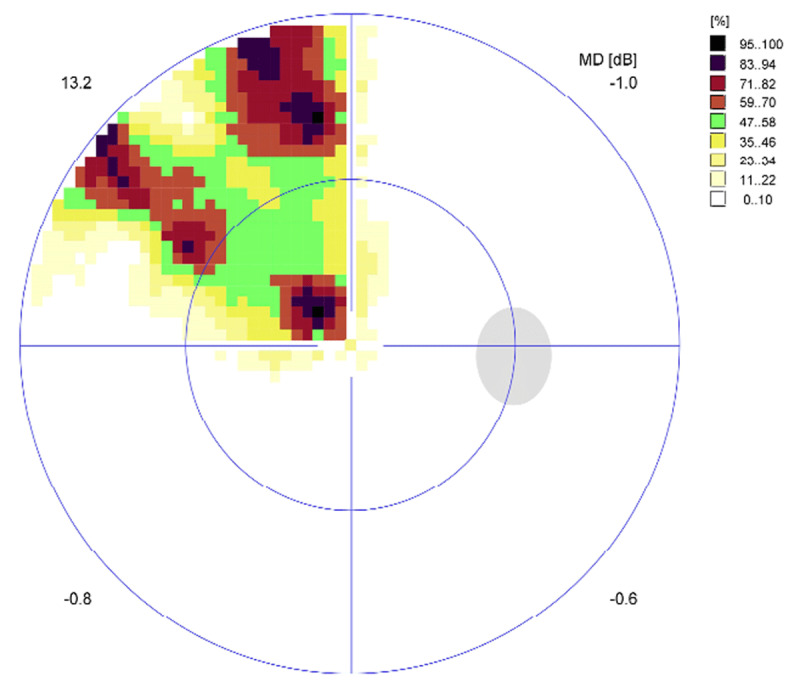
CS2 eight-week right eye threshold visual field test result. Representation of the central 30° of visual field area at eight weeks post-stroke.

**Figure 12:**
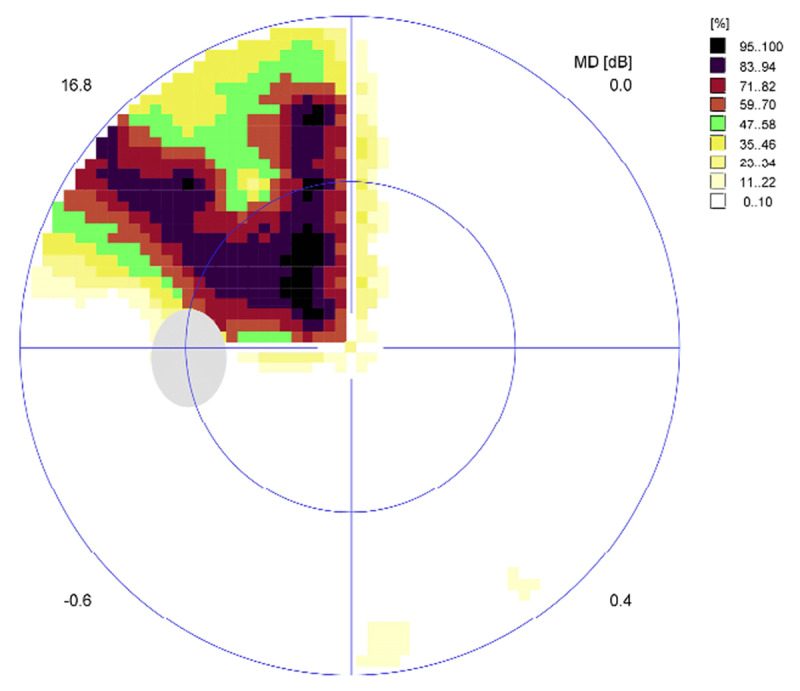
CS2 eight-week left eye threshold visual field test result.

At the eight-week assessment, CS2 demonstrated further improvement in his visual field with a completely normal visual field result on binocular Esterman perimetry testing (0.0% loss) (Figure 10). On the same day, this participant underwent threshold perimetry testing to ascertain any relative visual field defects that are not always detected by Esterman testing alone (Figure 11, Figure 12). Threshold testing demonstrated a residual visual field defect to the left side, which was particularly evident in the superior quadrant of both eyes.

**Figure 13:**
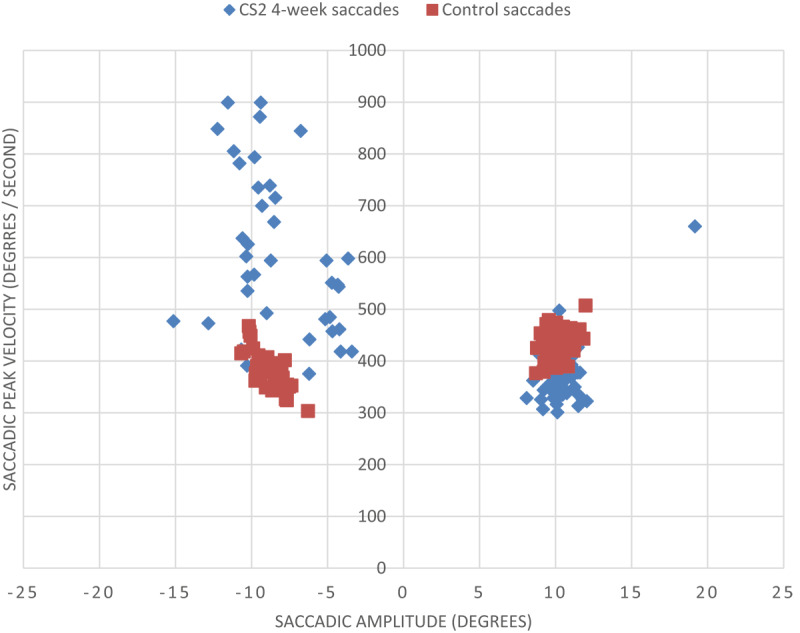
Saccadic peak velocity v saccadic amplitude for CS2 at four weeks and a control subject (minus values represent leftward saccades into hemianopic side).

Since his four-week assessment, he had continued to undertake visual scanning exercises daily for at least 30 minutes, which involved practising saccadic eye movements.

**Figure 14:**
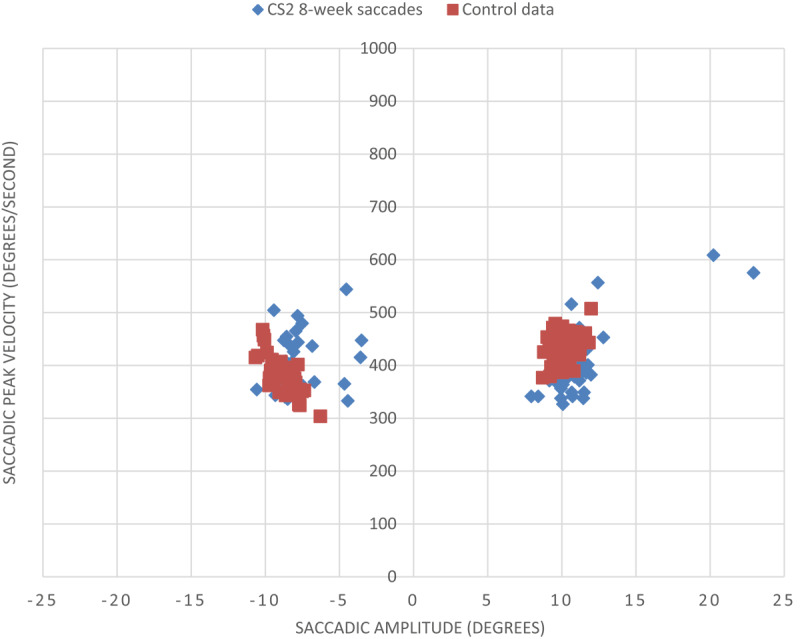
Saccadic peak velocity v saccadic amplitude for CS2 at eight weeks and a control subject (minus values represent leftward saccades into hemianopic side).

At the eight-week assessment, saccadic amplitudes were visibly more symmetrical between the right and left sides and more consistent with control saccadic parameters (Figure 14).

When comparing the CoV scores from this eight-week assessment to the previous four-week assessment, for amplitude the leftward CoV reduced from 35.6% to 22.2%, demonstrating less variation on what was previously the hemianopic side (Table 6). The rightward CoV increased from 14.6% to 21.8%, overall making the variations more symmetrical between sides.

When comparing velocity CoV scores, there were similar changes in variation scores from the four-week measurements, with a significant reduction in leftward CoV score (26.5% to 14.0%).

Despite reductions in variation, there was still more variation in scores between the right and left sides between the participant and age-matched control, suggesting an underlying saccadic deficit remained to some extent.

CS2 displays significant variation in the peak velocity measurements of his saccades and less accuracy to locate the target at its 10° location. This is more significant for his leftward saccades (the side affected by hemianopia) and is displayed by a large scattering of measurements to the left side. Saccades to the hemianopic side were faster and less accurate for this participant.

In contrast, the control subject displayed in Figure 13 makes accurate saccades to the target, which was presented at 10° to the right and left. The peak velocity shows a small variation with clustering of measurements around the same region.

There is a large improvement in the variation of saccadic parameters for participant CS2 at eight weeks post-stroke from the four-week measurements (as displayed in Figure 13).

## Discussion

The objective of measuring saccades in study participants was to investigate the relationship between adaptation to hemianopia and change in saccadic measurements over time. Due to limitations in target visualisation with hemianopic visual field loss, this objective was not fully achieved. The measurement of saccades over a period of time was limited to five participants. No calibrated saccade measurements at baseline were possible due to visual field restrictions. Other studies in the literature comparing saccadic parameters in hemianopic patients have overcome these limitation by fixing the head in place, awaiting recovery of visual field to allow standard calibration or allowing eye movements and hence not measuring visually directed reflexive saccades. Due to the acute and clinical nature of this study in an NHS setting, alternative research-based methods were not possible; hence, limitations were unavoidable.

For those participants who were able to visualise the target and therefore complete saccadic measurements, there was a difference between saccadic parameters to the hemianopic and the non-hemianopic sides, with some parameters being significantly different despite the small sample size. This finding of a discrepancy in saccadic parameters between the hemianopic and non-hemianopic sides is in agreement with previous studies for the task of visual scanning (Zihl 1995; Pambakian et al. 2000). For example, Pambakian et al. report a reduced amplitude for saccades directed towards the hemianopic side, in agreement with the four-week saccades in this sample (Table 4). This difference suggests an underlying saccadic deficit in these participants not explained by an absence of vision at the target position. It is possible that the difference in saccades to each side could be attributed to an adaptation of eye movements in hemianopia, or development of compensatory eye strategies, that occur at a different rate to visual field recovery. The discrepancy could also be due to specific damage within the brain pathways responsible for saccadic generation and control. Due to the limited brain scan information for participants in this acute onset stroke study, specific information regarding areas of brain damage was not possible.

One of the cases examined in more detail (CS2) showed an increased amplitude and peak velocity of saccades to the affected side, in contrast to the reported decrease. This supports the theory that people compensate for their visual field loss in different ways, using a wide range of strategies, making it difficult to ascertain the underlying mechanisms, especially with small sample sizes. Due to test limitations, it is not possible to make clinical recommendations based on these findings.

The type of visual field test used to assess patients is an important consideration for the assessment of hemianopia. Both of the case study participants in this research achieved a normal assessment on binocular Esterman visual field testing at eight weeks post-stroke but did in fact have a detectable defect on threshold testing. The binocular Esterman is a supra threshold test, meaning its results only provide information about absolute visual field defects (Rowe 1998). In addition, the binocular nature of testing means that fixation cannot be monitored during testing. However, the case studies analysed in detail displayed asymmetrical saccades to the right and left sides at four weeks and eight weeks post-stroke, despite being able to visualise the target shown and having only a minimal residual visual field defect. Although a relative visual field defect remained on threshold testing for both participants, absolute defects were not in the area of target presentation when viewing the targets with both eyes open (10° right and left on the horizontal plane). It has previously been reported that a saccadic deficit may simply be the result of a lack of awareness of a visual target at which to direct the saccade (Zihl 1995; Pambakian et al. 2000). The case studies reported here support the notion of differing rates of recovery for visual field loss and saccadic function. Furthermore, an underlying saccadic deficit with hemianopia is possible and may not be entirely related to target visualisation. This underlying saccadic deficit, if present, is likely to contribute to visual symptoms and difficulty with everyday activities such as reading and navigation. These defects are potentially undetected by routine visual field assessment. An improvement in this saccadic deficit using scanning training has the potential to improve how people adapt to hemianopia.

In summary, the saccadometer is a useful, if limited, instrument for the quantitative measurement of saccades in hemianopia. It can provide information on saccadic parameters following partial recovery of visual field loss and information on the presence of other oculomotor defects. The assessment of saccadic parameters can provide valuable information about subtle visual impairments noticed by people with hemianopia that are otherwise undetected by clinical assessment. Eye trackers, however, require calibration in a procedure that involves participants making saccades to visual targets. Therefore, whether accurate and meaningful information is obtainable with other eye trackers is doubtful, especially in an acute clinical setting.

Measurement of saccades was limited in this study due to the acute nature in which participants were assessed post-stroke and the research focus being on adaptation rather than recovery. An underlying saccadic deficit in hemianopia is suggestive of the hypothesis that saccadic improvement through training could aid adaptation; however, further research is required in this area. The observation of change in saccades over time was not possible due to measurement limitations, and only five participants were able to complete repeated measures. It is important to report these discovered calibration limitations, as there is little to no mention of such difficulties in the existing literature base and calibration is a vital procedure for the accurate recording of saccades.

The exploration of saccadic eye movements in relation to post-stroke hemianopia adaptation and response to saccadic training requires further examination. Considerations such as timing of assessment, use of measuring devices in an NHS acute setting and acceptability for acutely unwell stroke survivors will need to be considered for any future work.
